# Association between patterns of leisure time physical activity and asthma control in adult patients

**DOI:** 10.1136/bmjresp-2015-000083

**Published:** 2015-07-24

**Authors:** Simon L Bacon, Catherine Lemiere, Gregory Moullec, Gregory Ninot, Véronique Pepin, Kim L Lavoie

**Affiliations:** 1 Montreal Behavioural Medicine Centre, Hôpital du Sacré-Cœur de Montréal, Montréal, Québec, Canada; 2 Research Centre, Hôpital du Sacré-Cœur de, Montréal, Québec, Canada; 3 Department of Exercise Science, Concordia University, Montreal, Quebec, Canada; 4 Faculty of Medicine, University de Montreal, Montreal, Quebec, Canada; 5 Department of Psychoeducation and Psychology, University of Quebec at Outaouais (UQO), Saint-Jérôme, Quebec, Canada; 6 Laboratory Epsylon EA4556, University of Montpellier 1, Montpellier, France; 7 Department of Psychology, University of Quebec at Montreal (UQAM), Succursale Center-Ville, Montreal, Quebec, Canada

**Keywords:** Asthma, Exercise

## Abstract

**Background:**

Physical activity has been shown to have various health benefits in patients with asthma, especially in children. However, there are still limited data on the nature of the association between physical activity and asthma control in adults.

**Objective:**

The objective of the current study was to determine the nature of the association between physical activity and asthma control, with particular emphasis on the intensity of the activity and seasonal variations.

**Methods:**

643 adult patients with objectively confirmed asthma (mean age (SD)=53 (15) years, 60% women) were interviewed by telephone. Patients completed the asthma control questionnaire (ACQ), the asthma quality of life questionnaire, and a 1-year physical activity recall questionnaire to assess leisure time physical activity (LTPA).

**Results:**

Total LTPA was related to control (β (95% CI)=−0.013 (−0.030 to 0.006)), with those doing recommended levels of LTPA being nearly 2.5 times more likely to have good control compared with inactive patients. Analysis of seasonal exercise habits found that winter LTPA (β=−0.027 (−0.048 to −0.006)) was more strongly associated with ACQ scores than summer LTPA (β=−0.019 (−0.037 to −0.001)). Adjustment for age, sex, season of assessment, inhaled corticosteroid (ICS) dose, body mass index, and current smoking status reduced the strength of the relationships.

**Conclusions:**

Data indicate that higher levels of LTPA are associated with better levels of asthma control in adult patients with asthma, and that this seems to be more pronounced among asthmatics who do the recommended levels of exercise.

**Key messages**

- Engaging in optimal levels of recommended physical activity was associated with significantly better levels of asthma control in adults.
- Undertaking physical activity during winter, rather than summer, was a stronger predictor of better asthma control.
- This study provides further evidence of the potential benefits of physical activity as an adjunct treatment in adult patients with asthma.

## Introduction

The evolution of diagnostic and treatment strategies for asthma means that, in theory, all patients with asthma could be well controlled.^1^ ^2^ However, asthma remains poorly controlled in the majority of patients, for example, 60% of Canadian^3^ and 75% of European^4^ patients report having poor control. This inability to decrease asthma symptomology appears to be driving the global burden of asthma, and is associated with increased healthcare utilisation and greater functional impairment.^1^ ^5^ Identification of factors which might aid or improve patients’ control of their asthma over and above pharmacotherapy may help reduce the burden of asthma.

Increased levels of leisure time physical activity (LTPA) have been linked to improved health and health outcomes across a variety of chronic diseases,^6^ including asthma.^7^ ^8^ For example, increasing levels of LTPA in older women with asthma were associated with a decreased likelihood of reporting an asthma exacerbation over 1 year,^8^ and an intervention to increase LTPA was associated with a 12-month improvement in asthma control and quality of life.^9^ Additionally, physically inactive adults with asthma were more likely to be hospitalised overnight and seek multiple physician visits compared to active adults with asthma and active adults without asthma.^7^

Outside of asthma, it would seem that the impact of LTPA on health outcomes might differ as a function of different exercise intensities and patterns. For example, moderate-intensity aerobic exercise seems to be as beneficial for reducing blood pressure as high-intensity aerobic exercise,^10^ and there was a marked difference in activity levels as a function of season (lower levels in winter vs summer) in patients with diabetes.^11^ However, the role of different intensities and seasonal patterns of LTPA has not been previously assessed in adult patients with asthma. The identification of optimal patterns of LTPA for asthma control would be important for the development of future interventions.

The purpose of this study was to assess associations between LTPA (total activity and different seasonal patterns and intensities of activity) and levels of asthma control in a Canadian cohort of adult asthmatics.

## Materials and methods

### Study participants and procedure

This study was a subanalysis of an existing longitudinal cohort study assessing the impact of psychosocial factors on asthma^12^ ^13^ and included 643 adult patients with physician-diagnosed asthma who were interviewed at the first follow-up appointment. At baseline, patients with physician-diagnosed asthma^14^ were recruited from the outpatient asthma clinic of Hôpital du Sacré-Coeur de Montréal between June 2003 and January 2007. Eligible patients were between the ages of 18 and 75 years, and either French-speaking or English-speaking. Patients with occupational asthma, a significant comorbid medical condition (eg, cardiovascular disease, chronic obstructive pulmonary disease) or evidence of severe psychopathology (eg, schizophrenia) were excluded. For the follow-up interview, all patients were recontacted by phone. The follow-up interview included assessments of asthma control (verbal administration of the asthma control questionnaire (ACQ) and LTPA: verbal administration of the 12-month physical activity recall. In addition, patients were then sent a questionnaire pack to complete and return in a self-addressed envelope. Those patients who provided any follow-up data were included in the current analyses.

### Ethics statement

This project was approved by the Research Ethics Board of Hôpital du Sacré-Cœur de Montréal (#2003-10-198; 2010-95), and written consent was obtained from all participants.

### Measures

#### Asthma control questionnaire

The ACQ^15^ is a seven-item questionnaire that assesses levels of asthma control in the past week according to standard criteria specified by international guidelines,^1^ and yields a mean score out of 6. Six of the seven items are self-reported by the patient with one item (forced expiratory volume in 1 s (FEV1)% predicted) calculated from pulmonary function testing. As this questionnaire was administered over the phone, assessment of FEV_1_% predicted was not possible, so the current analyses reflects only the first six items (which has been shown to be a valid and reliable method of administering this instrument^16^). Previous research has shown that scores of ≥0.8 indicate poorly controlled asthma^17^ ^18^ and a change or difference of ≥0.5 in the ACQ has clinical significance.^19^

#### Asthma quality of life questionnaire

The asthma quality of life questionnaire (AQLQ) is a self-report questionnaire, consisting of 32 items, that assesses asthma-related quality of life across four life domains, symptoms, activity limitations, environmental stimuli and emotional distress, each of which may be affected by asthma,^20^ and yields a mean score out of 7. The AQLQ has demonstrated excellent measurement properties and has been validated in Canadian French,^21^ and was completed as part of the questionnaire pack that was mailed to participants.

#### Adapted 12-month physical activity recall interview

The 12-month physical activity recall interview (12M-PAR)^22^ ^23^ is a self-report questionnaire which assesses the average amount of LTPA that individuals perform over the course of a year. This scale includes activities at three intensity levels: moderate (eg, brisk walking), hard (eg, dancing) and very hard (eg, swimming), and generates estimates of energy expenditure (metabolic equivalent (MET)-h/week). Of note, 10 MET-h/week approximates to current guidelines for healthy activity levels of 30 min of moderate activity on most (ie, 5) days of the week.^23^ For this study, the questionnaire was administered over the phone and was adapted to ask about the summer (May–October) and winter (November–April) months separately, due to the extreme difference in temperature and weather conditions in Montreal.

#### Other asthma measures

All self-reported clinical data, including medical/asthma history, skin prick atopy status^24^ and medication dosage (including the inhaled corticosteroid (ICS) dose, which was used as a measure of severity^1^), were verified by medical chart review. In addition, baseline spirometry data were used to assess pulmonary function for descriptive purposes.

### Analyses

To ensure accuracy of data entry, all records were double entered and compared using PROC COMPARE (SAS). Missing data were imputed using PROC MI (SAS) and five independent data sets were generated.^25^ The specific variables which were included in the imputation and the amount of missing data are indicated in table 1. For the analyses, estimations for model coefficients were generated using PROC MIANALYZE according to Rubin's rules.^26^

**Table 1:** Demographic and medical/asthma characteristics presented as participating or not in LTPA

	No LTPA n=245 (38%)		Some LTPA n=398 (62%)				
	Mean or %	95% CI	Mean or %	95% CI	n	F	p Value
Demographics							
Age (years)*	53	51 to 55	54	52 to 55	643	0.12	0.732
Sex (% male)	40	34 to 46	39	34 to 44	643	0.07	0.798
Ethnicity (% white)	93	90 to 96	92	90 to 95	639	0.17	0.682
Cohabitating (% yes)	69	64 to 75	68	64 to 73	643	0.08	0.782
Employed (% yes)	52	45 to 59	47	42 to 53	589	1.17	0.280
BMI (kg/m^2^)*	**28**.**9**	**28.0** to **29.7**	**27**.**3**	**26.7** to **28.0**	**570**	**7**.**83**	**0**.**005**
Current smoker (% yes)	**13**	**9** to **17**	**6**	**3** to **9**	**582**	**8**.**22**	**0**.**004**
Asthma characteristics							
ACQ (0–6)*	**1**.**1**	**1.0** to **1.2**	**0**.**9**	**0.8** to **1.0**	**636**	**6**.**07**	**0**.**014**
Good ACQ (% yes)	**47**	**41** to **54**	**56**	**51** to **61**	**636**	**4**.**51**	**0**.**034**
AQLQ (1–7)*	5.3	5.1 to 5.4	5.4	5.3 to 5.5	582	2.85	0.092
Asthma duration (years)*	25	22 to 27	24	22 to 26	633	0.19	0.664
Atopic (% yes)	60	54 to 66	63	58 to 68	639	0.50	0.481
ICS dose (fluticasone equivalents)*	660	588 to 733	571	513 to 628	507	3.61	0.058
Bronchodilator use (# times in the past week)*	3.9	2.8 to 5.0	3.4	2.5 to 4.2	637	0.58	0.447

Bolding indicates values that are statistically significant.

*These values are reported as means.

ACQ, asthma control questionnaire; AQLQ, asthma quality of life questionnaire; BMI, body mass index; ICS, inhaled corticosteroid; LTPA, leisure time physical activity.

To assess the association between LTPA (continuous variable) and the asthma measures (ACQ and AQLQ scores), a series of general linear models were conducted. Logistic regression models were conducted to assess the relationship between a five-group LTPA variable (group 1=no LTPA and groups 2–5=4 quartiles of those doing any LTPA) and good asthma control (ACQ score ≤0.8). Finally, a series of exploratory analyses were conducted to assess the association between winter and summer LTPA, and moderate, hard and very hard LTPA (all as continuous variables) and ACQ score. All models were adjusted for age, sex, season of assessment and ICS dose, which were determined a priori. In order to examine the robustness of our findings, an additional series of analyses were conducted additionally adjusting for comorbid medical characteristics (current smoking and BMI) that have been associated with worse asthma outcomes^27^ ^28^ and that differed between those who did and those who did not engage in any LTPA. All tests were two-sided and the significance level was set at p<0.05. Data analysis was performed using SAS V.9.3 (SAS Institute, Cary, North Carolina, USA).

## Results

### Sample characteristics

The final sample of 643 patients included 387 (60%) women and they had a mean (SD) age of 53.4 (15.4) years. The mean (SD) duration of asthma for the sample was 20.0 (15.7) years. The mean (SD) (range) amount of LTPA was 4.8 (7.9) (0.0–48.1) MET-h/week, with 245 (38%) doing no LTPA at all. The mean sample (SD) (range) for ACQ and AQLQ scores was 1.0 (1.0) (0.0–5.2) and 5.4 (1.2) (1.0–7.0), respectively. Overall, these data are consistent with a tertiary care asthma population.

### Demographic and medical/asthma history characteristics

Demographic and medical characteristics as a function of having engaged in no LTPA versus any LTPA are presented in table 1. Relative to patients engaging in no LTPA, those doing any LTPA had a lower BMI and were less likely to be current smokers. With regard to asthma, patients engaging in some LTPA had better asthma control, tended to have better quality of life, and were prescribed less ICS.

### Association between LTPA and asthma control

As seen in table 2, unadjusted LTPA was negatively associated with ACQ score (β=−0.012). However, when adjusting for covariates, the association was no longer statistically significant. As seen in table 3, for analyses where LTPA was split into five groups, there were significant trends for higher levels of physical activity to be associated with better control. It should be noted that those in the highest quartile of physical activity were nearly 2.5 times more likely (ie, adjusted OR=2.47) to have good control compared with those who did not engage in any LTPA.

**Table 2:** Associations between different forms of leisure time physical activity and asthma control

	Unadjusted			Adjusted 1*			Adjusted 2†		
	β	(% CI) 95	p Value	β	(95% CI)	p Value	β	(95% CI)	p Value
Whole sample	**−0**.**012**	**(−0.022 to −0.002)**	**0**.**017**	**−**0.009	(**−**0.019 to 0.001)	0.068	**−**0.008	(**−**0.018 to 0.002)	0.115
Season									
Winter	**−0**.**027**	**(−0.048 to −0.006)**	**0**.**011**	**−0**.**022**	**(−0.043 to −0.001)**	**0**.**040**	**−**0.019	(**−**0.041 to 0.002)	0.072
Summer	**−0**.**019**	**(−0.037 to −0.001)**	**0**.**039**	**−**0.013	(**−**0.030 to 0.006)	0.151	**−**0.011	(**−**0.029 to 0.006)	0.207
Intensity									
Moderate	**−**0.011	(**−**0.037 to 0.015)	0.425	**−**0.010	(**−**0.035 to 0.016)	0.459	**−**0.008	(**−**0.033 to 0.018)	0.555
Hard	**−**0.052	(**−**0.137 to 0.033)	0.231	**−**0.044	(**−**0.130 to 0.041)	0.309	**−**0.040	(**−**0.126 to 0.045)	0.351
Very hard	**−0**.**012**	**(−0.024 to −0.001)**	**0**.**031**	**−**0.009	(**−**0.020 to 0.002)	0.114	**−**0.008	(**−**0.020 to 0.03)	0.170

Bolding indicates values that are statistically significant.

*Adjusted for age, sex, inhaled corticosteroid dose and season recruited in.

†Adjusted for age, sex, inhaled corticosteroid dose, season recruited in, body mass index and current smoking or not.

**Table 3:** Exercise, asthma control and asthma quality of life levels by quartiles of LTPA

	No exercise	Quartile 1	Quartile 2	Quartile 3	Quartile 4	Trend
n	245	99	100	99	100	
LTPA, Mean±SD (range) (MET-h/week)	0±0	1.0±0.4 (0.1–1.7)	2.8±0.7 (1.8–4.2)	7.1±1.9 (4.3–10.9)	20.1±8.9 (11.1–48.1)	
Total ACQ score† (0–6)	**1.10**	**0.92**	**1.03**	**0.92**	**0.84**	**p=0.037**
Predicted good control*	Ref	0.87 (0.38–2.00)	0.98 (0.43–2.22)	0.84 (0.37–1.91)	**2.47 (1.06–5.73)**	
Total AQLQ score† (1–7)	5.27	5.29	5.23	5.49	5.49	p=0.058

Bolding indicates values that are statistically significant.

All analyses adjusted for age, sex, ICS dose and season recruited in.

*Good control=ACQ score ≤0.8.

†As the analyses were conducted using multiple imputation, it is not appropriate to include SDs with the mean scores.

ACQ, asthma control questionnaire; AQLQ, asthma quality of life questionnaire; ICS, inhaled corticosteroid; LTPA, leisure time physical activity; MET, metabolic equivalent.

### Exploratory analyses between LTPA patterns and asthma control

As expected, participants generally engaged in more LTPA during the summer months compared to the winter months. We observed a significant negative relationship between winter and summer LTPA and ACQ scores (table 2), with winter exercise showing a stronger relationship to ACQ than summer exercise. However, in fully adjusted models, neither winter nor summer LTPA remained statistically significantly associated with control. When intensity of exercise was assessed separately, there was a statistically significant negative relationship between very hard LTPA and ACQ, but there were no relationships between moderate and hard LTPA and control (table 2). As with the seasonal variations, adjustment for covariates resulted in a reduction in the size of the effect for very hard LTPA, with a loss of statistical significance.

An additional analysis was conducted to see if there was a difference in ACQ scores according to the season of recruitment, for which there was none (β=0.055, 95% CI −0.105 to 0.215, p=0.50), indicating that those recruited during summer (mean=0.97) had similar ACQ scores to those recruited in winter (mean=1.02).

### Association between LTPA and asthma quality of life

LTPA was not significantly associated with AQLQ scores in unadjusted (β=0.011, 95% CI −0.001 to 0.024, p=0.08) or adjusted (β=0.006, 95% CI −0.006 to 0.019, p=0.30) analyses. However, it should be noted that the nature of the relationship between the two variables was in the hypothesised direction, that is, increased LTPA was associated with higher scores on the AQLQ. As seen in table 2, when LTPA was split into five groups, there was no significant association between LTPA and AQLQ scores.

## Discussion

This study assessed the cross-sectional association between LTPA and asthma control in a cohort of adult patients with asthma. Results showed that although patients engaging in higher levels of LTPA appeared to have better asthma control, this effect was lost once important covariates were adjusted for. In addition, those participants who engaged in the most amount of exercise (about 30 min of moderate exercise per day, most days of the week) were almost 2.5 times more likely to be well controlled compared with those who did not engage in any exercise, a result which held even with adjustment for covariates. Further interrogation of the data revealed that the amount of activity engaged in during the winter months was a stronger predictor of asthma control than summer activity.

In general, these findings are in line with the previous literature that has found decreases in asthma exacerbations and healthcare use in patients who engage in LTPA.^7^ ^8^ However, our study adds to the extant literature by assessing seasonal variations in activity and its association with asthma control. Asthmatics who exercised tended to perform more activity in the summer months than in the winter months, which is consistent with other studies.^29–31^ Although this result was unsurprising, the finding that winter activity was more strongly associated with control than summer activity was unexpected. We know of no studies that have found a differential relationship between physical activity and disease morbidity as a function of patterns of seasonal engagement. How engaging in physical activity over the winter may be associated with better control is not known. However, it is possible that asthmatics who continued to exercise during the winter may have accumulated more activity over the year, and that continuous and cumulative exercise may be a key element in the positive role of physical activity on asthma. Given the cross-sectional nature of the study, we cannot discount the possibility of reverse causality and that those with better control were able to exercise more during the winter. For example, it has been shown that for any specific intensity of exercise, there is a great decrease in FEV_1_^32^ and overall worse bronchial hyper-responsiveness^33^ in winter compared with summer. These, coupled with a greater probability of having exercise-induced bronchoconstriction during the colder, dryer months of winter,^34^ mean that there is the possibility of patients having generally poorer control in winter, which could then translate into less activity.

Further investigation into the factors which might account for the differential relationships between seasons is warranted. For example, patients with asthma who significantly reduced their exercise levels during the winter may have negative beliefs about exercise-induced symptoms (eg, increased risk of bronchoconstriction while exercising in cold temperatures), which could be driven by worsening bronchial obstruction and hyper-responsiveness (as detailed above), and/or an increase in perceived barriers to exercise (eg, concerns of the status of the roads, cost of membership to a fitness centre, etc). Understanding such facets may help to improve aspects of asthma treatment, for example, altered medication dosing in winter months, and self-management.

When interpreting the potential seasonal variations in the LTPA-asthma control relationship, one must be cognisant of the impact of weather changes on asthma in general. A number of studies have identified that asthma symptoms seem to be worse and asthma-related hospitalisations more frequent during winter relative to summer.^35–38^ Consistent with our findings, it may be that due to the higher symptom burden, exercising during winter is a more effective adjunct therapy to medications than during summer. Furthermore, there is some evidence that the severity of asthma-related hospitalisations may be higher during the summer,^35^ and it would seem that more asthma-related deaths occur during the summer compared with the winter.^39^ As such, in the context of our study, any benefits of engaging in LTPA on asthma during summer may not be related to asthma control but may be associated with a reduction in the severity of asthma exacerbations, which were not assessed in this study. While these studies may help explain the current findings, it should be noted that such seasonal variations may be less pronounced as patients get older, with minimal difference seen in seasonal variations in hospitalisations for middle-aged patients.^38^ ^40^ Given that our cohort was primarily middle-aged, this could account for the lack of seasonal difference in ACQ score for our study.

This study has some limitations that warrant discussion. First, we used a self-reported physical activity questionnaire to measure LTPA. However, such questionnaires have been shown to be reasonably consistent with other measures of activity.^29^ ^30^ Another limitation is that assessments of asthma control were conducted at only one time point; as such, season variations in LTPA and asthma control may not have been temporally assessed. However, we did not find seasonal differences in ACQ scores at recruitment and the season of recruitment was adjusted for in analyses. It should also be noted that the study was conducted in Montreal, which has a significant temperature gradient across the seasons, where winter can average −20°C and summer can be above 20°C. As such, it may not be possible to generalise these results to more temperate climates. Finally, this study is cross-sectional, so it is impossible for us to be able to define the exact direction of the relationship between asthma control and LTPA. As mentioned above, it is possible that our results reflect the possibility that those with better asthma control engage in more physical activity. However, recent studies have shown a prospective relationship between physical activity and a lower risk for asthma exacerbations, decreased healthcare usage, and improvements in quality of life.^7–9^

Despite some limitations, this study also has a number of important strengths. It was conducted with a well characterised sample of objectively diagnosed asthmatics. The sample size was relatively large and included both men and women.

## Conclusions

In summary, this study found evidence for an association between LTPA and asthma control in a large tertiary-care sample of Canadian adults with asthma, with recommended levels of LTPA being associated with better levels of asthma control. Furthermore, it would seem that engaging in LTPA during winter, rather than summer, was a stronger driver of the relationship. As this study was not designed to examine the mechanisms linking LTPA to asthma control, future studies should examine the pathways by which patients with asthma may benefit from exercising regularly across all seasons, but especially during the winter months. Finally, longitudinal and intervention studies are needed to determine the minimal dose of LTPA and the most cost-efficient strategies to improve asthma control in this kind of population.
